# Base editing and prime editing in laboratory animals

**DOI:** 10.1177/0023677221993895

**Published:** 2021-02-17

**Authors:** Federico Caso, Benjamin Davies

**Affiliations:** Wellcome Centre for Human Genetics, 6396 University of Oxford, UK

**Keywords:** Animal models, base editing, prime editing, adenine base editing, cytosine base editing, gene therapy, transgenesis, CRISPR

## Abstract

Genome editing by programmable RNA-dependent Cas endonucleases has revolutionised the field of genome engineering, achieving targeted genomic change at unprecedented efficiencies with considerable application in laboratory animal research. Despite its ease of use and wide application, there remain concerns about the precision of this technology and a number of unpredictable consequences have been reported, mostly resulting from the DNA double**-**strand break (DSB) that conventional CRISPR editing induces. In order to improve editing precision, several iterations of the technology been developed over the years. Base editing is one of most successful developments, allowing for single base conversions but without the need for a DSB. Cytosine and adenine base editing are now established as reliable methods to achieve precise genome editing in animal research studies. Both cytosine and adenine base editors have been applied successfully to the editing of zygotes, resulting in the generation of animal models. Similarly, both base editors have achieved precise editing of point mutations in somatic cells, facilitating the development of gene therapy approaches. Despite rapid progress in optimising these tools, base editing can address only a subset of possible base conversions within a relatively narrow window and larger genomic manipulations are not possible. The recent development of prime editing, originally defined as a simple ‘search and replace’ editing tool, may help address these limitations and could widen the range of genome manipulations possible. Preliminary reports of prime editing in animals are being published, and this new technology may allow significant advancements for laboratory animal research.

## Résumé

L'édition génomique par des endonucléases Cas programmables dépendantes de l'ARN a révolutionné le domaine de l'ingénierie génomique, en permettant de réaliser des changements génomiques ciblés avec une efficacité sans précédent et une application considérable dans la recherche sur les animaux de laboratoire. En dépit de sa facilité d'utilisation et de son application large, il reste des préoccupations quant à la précision de cette technologie et un certain nombre de conséquences imprévisibles ont été signalées, principalement en raison de cassure double-brin d'ADN induite par l’édition CRISPR conventionnelle. Afin d'améliorer la précision de l’édition, plusieurs itérations de la technologie ont été développées au fil des ans. L'édition de bases est l'un des développements les plus réussis, permettant des conversions de bases simples sans avoir besoin d'une cassure double-brin. L'édition de la base cytosine et de la base adénine est maintenant établie comme une méthode fiable permettant d'obtenir une édition précise du génome dans les études de recherche sur les animaux. Les éditeurs de bases de cytosine et d'adénine ont été appliqués avec succès à l'édition de zygotes, ce qui a entraîné la génération de modèles animaux. De même, les deux éditeurs de bases ont permis l’édition précise de mutations ponctuelles dans les cellules somatiques, facilitant ainsi le développement d'approches de thérapie génique. En dépit des progrès rapides réalisés au niveau de l'optimisation de ces outils, l'édition de bases ne peut traiter qu'un sous-ensemble de conversions de bases possibles dans une fenêtre relativement étroite et les manipulations génomiques plus importantes ne sont pas possibles. Le développement récent du « Prime Editing », initialement défini comme un simple outil d’édition par « recherche et remplacement », peut aider à résoudre ces limites et pourrait élargir la gamme des manipulations génomiques possibles. Des rapports préliminaires sur le « Prime Editing » chez les animaux sont en cours de publication. Cette nouvelle technologie pourrait permettre des progrès importants pour la recherche menée sur les animaux de laboratoire.

## Abstract (de)

Genom Editing durch programmierbare RNA-abhängige Cas-Endonukleasen hat das Genom-Engineering revolutioniert und ermöglicht gezielte genomische Veränderungen mit höchster Effizienz, die in der Versuchstierforschung in großem Umfang eingesetzt werden. Trotz der einfachen Handhabung und des breiten Anwendungsspektrums gibt es nach wie vor Bedenken hinsichtlich der Präzision dieses Verfahrens. Es wurde zudem über eine Reihe unvorhergesehener Folgen berichtet, die vorwiegend auf den DNA-Doppelstrangbruch zurückzuführen waren, den das herkömmliche CRISPR-Editing induziert. Um die Präzision des Editing zu verbessern, wurden im Laufe der Jahre mehrere Iterationen des Verfahrens entwickelt. Eine der erfolgreichsten Entwicklungen ist das Base Editing, bei dem einzelne Basen verändert werden können, ohne dass es zu einem Doppelstrangbruch kommt. Cytosin- und Adenin-Base-Editing haben sich mittlerweile als zuverlässige Methoden etabliert, um in Tierversuchsstudien eine präzise Genom-Editierung zu erreichen. Sowohl Cytosin- als auch Adenin-Basen-Editoren wurden erfolgreich für das Editing von Zygoten eingesetzt, um Tiermodelle zu generieren. In ähnlicher Weise haben beide Basen-Editoren das präzise Editing von Punktmutationen in somatischen Zellen erreicht, was die Entwicklung von Gentherapieansätzen erleichtert. Trotz enormer Fortschritte bei der Optimierung dieser Werkzeuge kann Base Editing nur eine Teilmenge möglicher Basenumwandlungen innerhalb eines relativ engen Fensters adressieren, und größere genomische Manipulationen sind nicht möglich. Die jüngste Entwicklung von Prime Editing, das ursprünglich als einfaches Editierwerkzeug für „Suchen und Ersetzen “definiert war, könnte dazu beitragen, diese Einschränkungen zu überwinden und das Spektrum der möglichen Genom-Manipulationen zu erweitern. Erste Berichte über Prime Editing bei Tieren wurden bereits veröffentlicht, und dieses neue Verfahren dürfte demzufolge bedeutende Fortschritte in der Versuchstierforschung ermöglichen.

## Resumen

La edición genómica mediante endonucleasa Cas dependiente del ADN ha revolucionado el campo de la ingeniería genómica, consiguiendo así un cambio genómico buscado con unas ineficiencias sin precedentes y con una aplicación significativa en la investigación con animales de laboratorio. A pesar de su uso sencillo y de su aplicación generalizada, sigue habiendo dudas sobre la precisión de esta tecnología y se ha registrado un número de consecuencias impredecibles, principalmente debido a la ruptura de la doble cadena de ADN a la que induce la edición CRISPR convencional. Para mejorar la precisión de la edición, se han desarrollado diferentes iteraciones de la tecnología durante años. La edición base es uno de los desarrollos más exitosos, que permite conversiones de base única pero sin la necesidad de una ruptura de la doble cadena. La edición base de citosina y adenina ahora se considera como un método fiable para conseguir una edición genómica precisa en los estudios de investigación con animales. Los editores base de citosina y adenina se han aplicado satisfactoriamente para la edición de zigotos, lo que ha llevado a la generación de modelos animales. Del mismo modo, ambos editores base han conseguido una edición precisa de mutaciones puntuales en células somáticas, lo que ha facilitado el desarrollo de métodos de terapia génica. A pesar del rápido avance de la optimización de estas herramientas, la edición base solo puede tratar un subgrupo de conversiones base posibles dentro de una ventana relativamente estrecha y unas manipulaciones genómicas más grandes no son posibles. El reciente desarrollo de la edición principal, inicialmente definida como una simple herramienta de «búsqueda y reemplazo», puede ayudar a tratar estas limitaciones y podría ampliar el rango de manipulaciones genómicas posibles. Se están publicando informes preliminares de edición principal con animales y esta nueva tecnología puede que permita unos avances significativos para la investigación con animales de laboratorio.

The discovery of CRISPR/Cas systems has had a substantial impact on our ability to modify the genome of laboratory animals.^1,2^ The CRISPR system as a laboratory tool is generally comprised of two elements: a Cas nuclease and a single guide-RNA (sgRNA), the first (typically 20) nucleotides of which define the genomic target site. Several other Cas proteins from different bacteria and archea have been identified over the years,^3^ each with its own size and characteristics; however, the most commonly used enzyme is *Streptococcus pyogenes* Cas9. When introduced into a cell, the Cas nuclease is guided by its sgRNA and introduces a double-strand break (DSB) at its target site. Repair of the DSB by non-homologous end joining (NHEJ) can lead to disruption of the target sequence (knock-out) through small insertions or deletions (indels) at the repair junction,^4,5^ Alternatively, by providing a repair template, the homology-directed repair (HDR) pathway can be used to introduce specific sequences into the genome (knock-in).^5^ Target sites are defined by the presence of a so-called protospacer adjacent motif (PAM), which is usually a 2–4 bp motif, depending on the Cas protein used. This restriction does not frequently impose any major limitations and CRISPR sites near mutagenesis targets are generally easily found.

**Figure 1.**
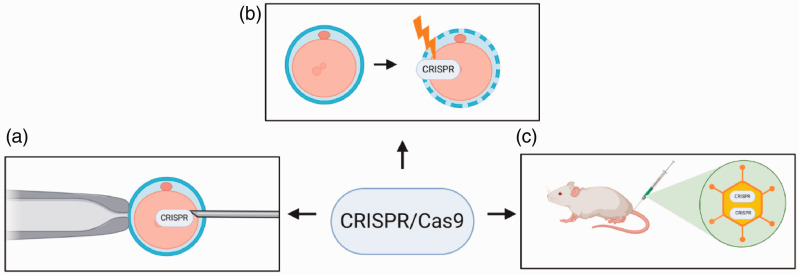
The most common delivery methods used for genome editing in laboratory animals. (a) The delivery of the CRISPR/Cas9 reagents into a zygote by microinjection. The embryo is held using a holding pipette (left) while the nuclear membranes and/or cellular membranes are pierced by a microinjection pipette, injecting the components directly into the cytoplasm or the nucleus. (b) Delivery by electroporation: low electrical current is used to increase cellular permeability, permitting the CRISPR/Cas9 reagents entry into the zygote. (c) Viral delivery of CRISPR/Cas9 reagents into somatic cells. An attenuated virus is equipped with a construct encoding the CRISPR/Cas9 reagents and inoculated into the organism, where the virus by infecting the host’s cells will also deliver the construct.

Over the years, techniques have been developed to deliver the CRISPR/Cas system into laboratory animals for the generation of genetically modified animal models. Two of the most common methods for delivery of Cas9 into fertilised zygotes are microinjection and electroporation. While the first relies on the physical injection of Cas9 into the zygote (Figure 1a),^6^ the latter uses pulses of electrical current to increase the permeability of the cell membrane, allowing Cas9 delivery into the zygote (Figure 1b).^7^ The early reports of Cas9 delivery to zygotes used Cas9 mRNA, whereas, more recently, ribonucleoprotein (RNP) preparations of Cas9 protein complexed with its sgRNA are more routinely used. CRISPR/Cas systems can also be introduced into somatic tissues of the live animal, commonly by viral delivery, where DNA sequences can be manipulated in the targeted cell type (Figure 1c).^8^

Although knock-out alleles can be made simply by relying upon NHEJ, generation of knock-in alleles with a specific DNA sequence modification can be more challenging. Firstly, the NHEJ repair mechanism is more dominant, resulting in a low generation efficiency of knock-in alleles, as well as the frequent introduction of indel mutations accompanying the knock-in allele. Moreover, the repair of DSBs has also been shown to result in large chromosomal deletions and rearrangements,^9–12^ suggesting that the introduction of DSB lesions are disadvantageous for precision engineering. Secondly, while NHEJ is active at any time, HDR occurs only during the G2 and S phase of the cell cycle,^13^ making insertion of exogenous DNA more difficult in non-dividing cells. Over the years, CRISPR systems have been developed to overcome these limitations. One of the more exciting advances in the field is base editing, followed more recently by prime editing. These methods avoid making the DSB lesion and could provide a safer means of genome engineering. In this review, we introduce these systems, and their advantages and disadvantages will be discussed in relation to their use in laboratory animal research.

## Base editing

**Figure 2.**
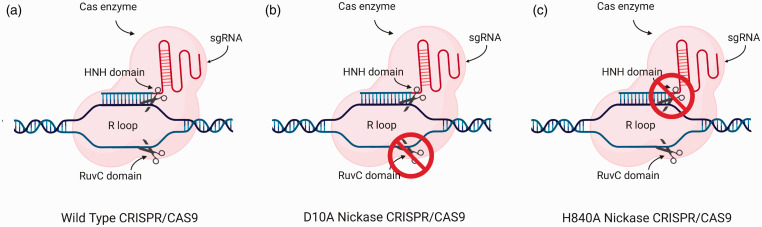
Wild type and nickase CRISPR/Cas9. (a) In the wild-type CRISPR/Cas9 system, the binding of the sgRNA to the target strand results in the formation of an R loop, and the two nuclease domains (HNH and RuvC) of the Cas9 enzyme are responsible for the cleavage of each single strand of DNA. (b) An engineered CRISPR/Cas9 nickase harbouring a D10A mutation that inactivates the RuvC domain, resulting only in the cleavage of the target strand of DNA (a single ‘nick’) via the still active HNH domain. (c) An engineered CRISPR/Cas9 nickase harbouring an H840A mutation that inactivates the HNH domain, resulting only in the cleavage of the non-target strand of DNA (a single ‘nick’) via the still active RuvC domain.

Base editors rely on a modified CRISPR system for the chemical conversion of a single base into another. Using a mutant Cas nuclease – a so-called nickase – in which one of the two catalytic domains has been inactivated (Figure 2), only one strand of DNA is cut; hence, the base editors are not able to introduce a DSB, avoiding many of the negative consequences resulting from this lesion. The mutant Cas protein is attached covalently to enzymatic domains that catalyse the conversion of a DNA base. In combination with a sgRNA directing the base editor to its target site, these enzymatic domains act locally to convert target bases in the vicinity. Their ability to target point mutations precisely makes them an ideal tool for modelling the effects of human single pathogenic variants in laboratory animals.^14^ The classical base editors are only able to convert bases within the same class, i.e. purine or pyrimidine, and are thus able to induce base transitions. Interestingly, a recent publication has introduced an editing system that allows C-to-G base transversions.^15^ Although not yet tested in animal models, this report extends the range of possible manipulations achievable with base editors.

## Cytosine base editing

**Figure 3.**
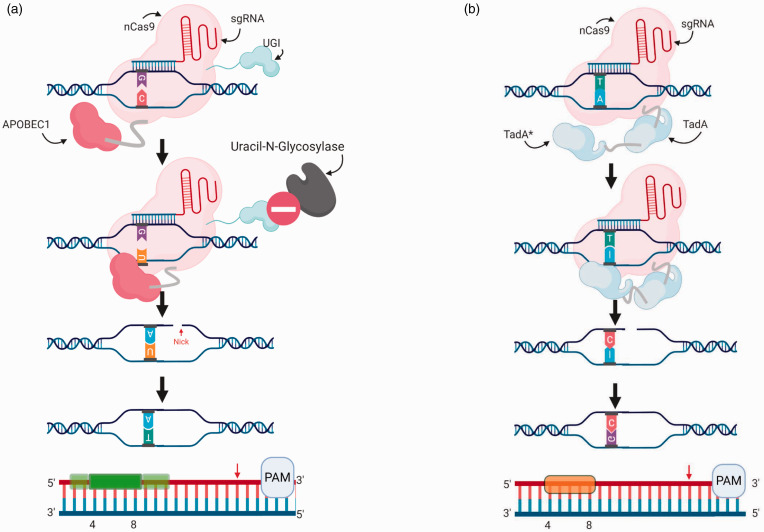
Base editing of DNA using cytosine base and adenine base editors. (a) The cytosine base editor, BE3, is shown binding to the sequence of interest and the process of cytidine deamination by APOBEC1 of the non-target strand after the DNA denaturation has been induced by the nCas9. The complete conversion of the base pair is then carried out by the DNA repair mechanism following the nick of the target strand, with the UGI inhibiting the UNG repair mechanism. A map depicting the activity window of BE3 in relation to the PAM site is shown at the bottom. (b) The adenine base editor, ABE7.10, is shown binding to its sequence of interest and the process of adenine deamination is carried out by the TadA*-TadA heterodimer on the non-target strand, following the DNA denaturation induced by the nCas9. The complete conversion of the base pair is then carried out by the DNA repair mechanism following the nick of the target strand. A map depicting the activity window of ABE7.10 in relation to the PAM site is shown at the bottom.

The first reported base editor was engineered to deaminate the exocyclic amine of the cytosine base. This causes the conversion of cytosine into uracil, which is then read as thymine by the DNA replication machinery.^16^ The ability to induce C-to-T changes within DNA is useful with respect to modelling human pathogenic genetic variants, where 14% of variants are caused by this class of transition.^17,18^ BE3 and BE4 are versions of the cytosine base editors (CBE) that are most commonly used.^14,16^ The central structure of these editors is composed of a Cas9 nickase (nCas9, D10A mutant; Figure 2b) fused to the APOBEC1 enzyme and one or two uracil glycosylase inhibitors (UGI). APOBEC1 is a mammalian cytidine deaminase, which interacts only with single strands of DNA.^19^ Directed to its target sequence by a sgRNA, the nCas9 opens the two DNA strands. The APOBEC1 can then deaminate any cytosine within an activity window on the non-target strand. This window is ∼5 bp long and lies ∼15 bp upstream of the PAM sequence. Once the cytosine has been converted into uracil, the UGI component inhibits the uracil-N-glycosylase (UNG) repair mechanism that would otherwise eliminate the mismatched U–G.^20^ Finally, the nCas9 causes a nick of the target strand. The nick encourages repair of the mismatched U–G pairing using the base-edited non-target strand as a template, inserting an adenine opposite the uracil. Upon DNA replication, the U–A pair is then converted to T–A, completing the conversion (Figure 3a).

Of note, a similar CBE system was simultaneously reported, which, instead of APOBEC1, uses the activation-induced cytidine deaminase (AID),^21^ an essential enzymatic domain involved in the hypermutation process of B cells in mammals. This system, named Target-AID, induced base editing in vitro, with efficiencies closely resembling BE3, making the two systems interchangeable but with differences in the editing window.^14,21^

## Applications of cytosine base editing: animal models

The successful creation of the first animal model through cytosine base editing was achieved shortly after publication of the system.^22^ Knock-out mutations in both the *Dmd* and *Tyr* genes were achieved by editing of C-to-T within a coding exon, creating a premature stop codon. Microinjection of BE3 mRNA and sgRNA into mouse embryos resulted in 55% of founder mice carrying mutations of the target *Dmd* gene. However, despite C-to-T conversion being the most common mutagenic outcome, alleles were generated that frequently showed additional mutations, including deletions at the target site.^22^ Additional studies, using the same microinjection technique, also reported successful mutagenesis, but similarly found indel and additional mutations to be a common occurrence.^23,24^ Base editing of the *Tyr* gene was also achieved via electroporation of mouse zygotes with a BE3 RNP.^22^ All of the resulting mice from this latter study harboured *Tyr* mutations, including two homozygous founders that showed a clear phenotype, demonstrating the efficacy of this delivery method. BE3 RNP was also used for the successful editing of *Tyr* in embryos of *Xenopus laevis*. C-to-T conversion at the *Tyr* target site was achieved at a rate of up to 20%, demonstrating functionality of this method beyond the mammalian system.^25^ Functionality of the Target-AID system in non-mammalian animal models was also demonstrated by successful editing of the *chd* and *oep* genes in zebrafish.^26^

The induction of precise base conversions has facilitated the generation of animal models harbouring mutations orthologous to those associated with human diseases, allowing for a better understanding of the underlying pathophysiology. Microinjection of mouse zygotes with BE3 mRNA and sgRNA led to the generation of *Psen1* mouse models, carrying pathogenic mutations associated with Alzheimer’s disease.^27^ Targeted base conversion occurred in 63% of the founder mice; however, due to the presence of multiple target cytosine residues within the targeting window, only 5% of the mice carried the desired substitution. This is a general issue with base editors as the presence of multiple cytosine residues can confuse outcomes. In addition, some indel mutations and incorrect conversions (base conversions that do not follow the canonical C-to-T) were observed. Similar results were also reported using CBEs in other animal model species, such as zebrafish and rabbits, indicating that BE3 has a high editing efficiency but the additional C-to-T conversions within the target window can lead to undesired alleles.^28,29^

Early studies in mouse zygotes demonstrated that CRISPR-induced mutation via conventional cutting and repair could be multiplexed, allowing several genes to be mutated in parallel. Successful multiplexing of the CBE system was also demonstrated by targeted knock-out of three auditory cell genes (*vGlut3, Prestin* and *Otoferlin*) by microinjection in mouse zygotes.^30^ All but one of the founder mice were found to be triple knockouts without any off-target mutations. Successful mutagenesis of two linked loci in mouse was also demonstrated by zygote microinjection of CBEs, avoiding the *cis* deletions that frequently occur when using conventional CRISPR editing.^31^ Successful multiplex editing was further reported in large animal models, with founders generated with the desired mutations at three target sites in pig,^32,33^ and in cynomolgus monkey.^34^ Although some incorrect conversions and deletions were reported, some founders carried homozygous edits of all three genes and displayed a clear phenotype.^32,34^ These studies confirm that the efficiency of the CBEs is sufficiently high to allow both simultaneous editing of multiple genes and the generation of homozygous alleles, thus obviating the need for further breeding. These impressive results can be considered as feasibility studies for base editing technology to be used for modelling multigenic disorders in animal models.

As an alternative to direct zygote injection, base editing can be combined with somatic cell nuclear cloning, which may provide an alternative methodology of production, particularly for large animal species. Indeed, the Dystrophin gene, *DMD*, along with *RAG1*, *RAG2* and *IL2RG* were successfully edited using CBEs in pig foetal fibroblasts, and these cells were then used as nuclear donors in cloning procedures, allowing the generation of base-edited pigs.^32^ Similarly, a pig fibroblast clone harbouring loss-of-function *GGTA1*, *B4galNT2* and *CMAH* alleles, generated in multiplex using a CBE, was also used successfully as a nuclear donor, allowing the generation of a pig model with ablated major hyperacute rejection-related xeno-antigen, paving the way for xenotransplantation research.^33^

## Applications of cytosine base editing: somatic cell editing in vivo

BE3 is also suitable for mutagenesis of somatic cells by local delivery into tissues, providing a preclinical model of how this technology might be adapted successfully for gene therapy. Local injection of BE3 RNP, packaged in a cationic liposome, into the mouse inner ear resulted in the introduction of a stabilizing mutation within the β-catenin gene.^35^ This led to modulation of Wnt signalling and an induction of mitotic division in cochlear support cells. Conventional editing by HDR failed to achieve this effect due to significantly more indels occurring at the target site, suggesting that BE3 editing was a safer system for somatic cells modification, avoiding the collateral damage occurring when introducing a DSB into the target site. Interestingly, the study determined that RNP delivery resulted in higher editing precision compared with plasmid delivery. The efficiency of delivering base editors as RNP was further corroborated in zebrafish.^36^

Not all tissues are amenable to direct delivery, subsequently viral delivery is a practical means of BE3 delivery to diverse tissue types and has been explored in animal models. The large BE3 machinery can be packaged in a single adenoviral vector and this was used to achieve a knock-out of the *Pcsk9* gene in mouse liver cells through retro-orbital injection. Viral delivery led to significantly reduced plasma PCSK9 protein levels, and the loss of this protein convertase led to a 28% reduction of plasma cholesterol – its predicted therapeutic effect. Deep sequencing confirmed a median editing efficiency of 24% and a ∼1% indel rate, which is far lower than the ∼40% rate observed in similar studies using conventional CRISPR editing.^37,38^ Although no off-target mutations were observed, a low rate of incorrect conversions at the target site was noted.^39^ Similarly, *in vivo* base editing successfully edited both mouse *Pcsk9* and the human gene within a humanised knock-in mouse, resulting in significantly lower plasma cholesterol.^40^ Finally, adenovirus-delivered BE3 has proven effective in safely editing *Pcsk9* prenatally.^41^ The proportion of edited alleles in foetal liver ranged from 10% to 15%, with an indel rate of only 2%, considerably lower than the indel rate observed using conventional CRISPR editing (∼40%). In addition, the same study elegantly rescued the lethal phenotype caused by *Fah* loss of function, which causes hereditary tyrosinemia type 1, by delivering BE3 targeting the *Hpd* gene to the foetus. Loss of function of *Hpd*, a gene acting upstream of *Fah* in the tyrosine catabolic pathway, prevents the accumulation of toxic metabolites and alleviates the *Fah* deficit. The resulting mice at 1 and 3 months of age showed 37% and 40% base editing efficiency, respectively, with no evidence of mutations in other organs, and achieved a better therapeutic outcome than conventional drug treatment.

While adenoviral vectors have proven effective for gene therapy, they have also induced significant immune response in the host. Adeno-associated virus (AAV), which evokes a milder immune response, is a frequently used alternative vector for gene therapy. AAV persists primarily as an episome – significantly, even in non-dividing cells – and a range of natural serotypes is available with different tissue tropisms.^42^ Due to its large size, BE3 cannot be packaged as a single AAV, but studies have achieved successful somatic cell editing by splitting BE3 into two vectors. This approach was used to correct a mutated *Pah* gene in mouse hepatocytes *in vivo*.^43^ Within weeks of treatment, corrected hepatocytes were found to increase from 10% to 25%, while 63% of the *Pah* mRNA was found to be edited correctly after injection of a higher BE3 dose. Although indel mutations were also observed, the effectiveness of the treatment was evident by an increase in weight in the experimental mice. Editing of mice astrocytes to achieve inactivation of a mutant *Sod1* gene for the treatment of amyotrophic lateral sclerosis (ALS) was achieved by intrathecal injection of split BE3 AAV, resulting in delayed progression of the disease in treated mice.^44^ Finally, split AAV was used successfully to deliver CBEs designed to correct a pathogenic mutation at the *Tmc1* gene associated with hearing loss, to the inner ear of mice.^45^ The mutant sequence was corrected at efficiencies of around 51%, restoring the morphology and sensory transduction of the inner ear hair cells, and, importantly, transiently rescuing aspects of the hearing deficit. Somatic mutations by BE3 were also elegantly shown to allow the efficient generation of breast cancer models in the mouse.^46^ A mouse model conditionally expressing BE3 in breast epithelia was generated, and sgRNAs inducing specific oncogenic mutations in *Akt1* and *Pik3ca* were then delivered by lentivirus *in vivo*. The results of these studies provide compelling evidence for an application of cytosine base editing as a tool for gene therapy in the future.

## Adenine base editors

Despite the utility of C-to-T (or G-to-A) mutations achievable by CBE, the base conversion that could correct A-to-G (or T-to-C) could result in substantial therapeutic potential as it has been calculated that 47% of all of human pathogenic mutations, could be addressed using such a tool, if PAM and activity-window constraints could be overcome.^14,17^ This requirement led to the development of a tool able to deaminate adenine, converting it into inosine.^47^ No adenine deaminases acting on single-stranded DNA are found in mammals and thus to overcome this obstacle, the Liu laboratory molecularly evolved an *Escherichia coli* tRNA adenosine deaminase (TadA).^48^ A mutated TadA* was generated through several rounds of bacterial selection aimed at uncovering protein variants associated with increased editing efficiency. The final version of the TadA* was paired to a natural TadA, which provides support as a docking station. The resulting optimised adenine base editor (ABE), known as ABE7.10, is composed of the normal TadA, the mutated variant TadA* and Cas9 nickase (nCas9, D10A mutant; Figure 2b). The system targets the sequence of interest using the sgRNA, where the nCas9 then separates the two strands, allowing the TadA* to deaminate all the adenines within an activity window on the non-target strand. The nCas9 induces a nick of the target strand, encouraging its repair using the edited strand, inserting a cytosine opposite the inosine, which, upon DNA replication, is then converted to a C–G pair, completing the conversion. This system is characterised by a ∼4 bp window of activity ∼12 bp upstream of the PAM sequence where the TadA* can perform the conversion of any adenine within this window (Figure 3b).^47^

## Applications of adenine base editing: animal models

The ABE system was quickly tested for the efficient generation of animal models by introducing the ABE7.10 system into fertilised mouse zygotes as mRNA. Generation of *Tyr* and *Dmd* knock-out mice was achieved by targeting splice sites. Editing of the *Tyr* target site was achieved at 56% efficiency, while mice harbouring mutations at the *Dmd* gene were obtained at an efficiency ranging from 42% to 70% depending on the sgRNA used.^49^ Similar results were also reported at the *Tyr* gene,^50^ with base substitutions recorded in 78% of the edited mice. Moreover, no unwanted mutations or incorrect conversions (base conversions that do not follow the canonical A-to-G) were found at the *Tyr* site and no off-target mutations were detected across over a million candidate sites. Finally, mutant mice, albeit with a mosaic outcome, harbouring either androgen receptor (*Ar*) and homeobox13 (*Hoxd13*) gene mutations were achieved in 81–100% of the founder mice.^51^ A number of studies confirmed high editing efficiency in other animal species, such as rats and zebrafish, after appropriate optimization.^52–54^ Multiplex editing has also proven feasible with ABEs – a study in cynomolgus monkeys achieved targeted mutations at the *HBB* and *TP53* genes at a rate of 38.5% using microinjection of ABE7.10 mRNA and two sgRNAs.^34^ Taken together these studies prove the efficacy of adenine base editing for the genetic modification of several model species on a par with cytosine base editing.

## Applications of adenine base editing: somatic cell editing in vivo

Adenine base editing has also been proven to be useful for the mutation of somatic cells.^50^ In this study, the large size of the ABE7.10 construct (6.1 kb) also required the system to be split between two AAVs. The two vectors were delivered through intramuscular injection to target *Dmd* mutated muscle fibres in a mouse model of Duchene muscular dystrophy. The required conversion was found with a frequency of ∼3% without any indels or off-target mutations. This level of correction induced a ∼17% increase in dystrophin protein, where a 4% improvement is sufficient to relieve the symptoms of the disease. Similarly, correction of a pathogenic mutation in the *Fah* gene was achieved by hydrodynamic tail-vein injection of a codon-optimised ABE plasmid, rescuing the negative weight loss in the affected mice.^55^ Finally, an engineered RNA-encoded ABE, encapsulated into a lipid nanoparticle, was delivered into mouse livers via tail vein injection and achieved successful editing of *Fah* mutations, with an average DNA correction rate of 12.5%, resulting in a significant recovery of the treated mice.^56^ These preliminary studies make a compelling case for the future application of ABE in the field of gene therapy.

## Comparison between CBE and ABE

As detailed above, CBE and ABE systems have proven suitable for animal model generation, but have also opened up possibilities for a role in therapeutic interventions. However, the studies have revealed that the precision required for a conversion of a single targeted base is at times inadequate. Incorrect mutations can be caused either by the wrong target base being converted within the activity window, an incorrect base conversion or by indels. Several studies have compared the two base editor systems in this regard.^57,58^ CBE and ABEs showed broadly similar ranges of editing efficiencies within their activity windows, but a striking difference in the occurrence of unwanted mutations was found between the two editors. Depending on the CBE used, between 6% and 60% of edited alleles showed unwanted products such as indels, conversions of bases on the incorrect strand or conversions of the C base into either A or G. In contrast, 2% or less of ABE-edited alleles showed the aforementioned range of unwanted conversions. Strikingly, a 12% indel incidence was observed for BE3, with this percentage falling to 0% for ABE7.10.^57^ With respect to off-target mutations, a follow-up study from the same laboratory used whole genome sequencing on trios of animals (offspring and parents) and found considerably higher levels of non-specific mutation when using the CBE, BE4 as opposed to ABE7.10 and, importantly, this study used the same sgRNA sequence for both editors in a carefully controlled comparison.^58^

Although these studies demonstrated significantly higher accuracy of ABE, both systems display a ratio of correct edits to unwanted mutation that is improved significantly when compared with the ratio usually characterising HDR by conventional CRISPR editing.^35^ These results were further corroborated by comparing the editing efficiency of CBE (BE3) and ABE (ABE7.10) in rabbits.^29^ A slightly higher targeted editing efficiency and a lower off-target rate was observed for ABE7.10, compared with BE3, with no indel generation observed when using ABE7.10. The study also found that a series of unwanted base conversions that occurred when using CBEs were not observed when ABEs were used. However, the study also highlighted how HDR by conventional CRISPR editing is still less effective than both CBE and ABE.

Moreover, the elegant application of a modified Digenome-seq test to detect off-target sites genome-wide, following the use of BE3 and ABE7.10, unveiled important information on the difference in off-target sites caused by base editors and conventional Cas9.^59,60^ Notably, the studies highlighted the uniqueness of the off-target sites for each editing system employed. Finally, an in-depth study on the off-target rates of CBE, ABE and conventional CRISPR editing relying on HDR was carried out by comparing the single nucleotide variant (SNV) differences between the edited and unedited nuclei of blastomeres following microinjection of the systems.^61^ The study reported that neither CRISPR/Cas9 nor ABE generated more SNVs than were found in the wild-type cell, while BE3 produced a staggering average of 283 SNVs per embryo. Interestingly, this occurred only when sgRNA was not provided and, notably, over 90% of the conversion were both G-to-A and C-to-T, suggesting a low specificity of BE3 in the absence of a sgRNA. These results indicate that BE3 is the editing system with the highest off-target rate. It is believed that the unmutated APOBEC1 employed in BE3 conversions is responsible for the high rate of unwanted editing.^61,62^

Based on these studies it appeared that adenine base editing was far more accurate than cytosine base editing. However, ABEs have been shown to induce cytosine to guanine conversions with a rate of up to 11%, and the presence of a TC*N nucleotide motif (a triplet of three bases composed of thymine, cytosine and a pyrimidine base) within the editing window was suggested to be a possible trigger for this aberrant editing.^63^ Although significantly lower compared with the canonical adenine deamination, unwanted C-to-G conversion has to be taken into consideration when designing animal experiments using these tools.

In addition to off-target mutations on DNA, two recent investigations demonstrated a high rate of off-target activity in the transcriptome of base-edited genomes, caused by direct deamination of the RNA, raising more accuracy concerns. Firstly, BE3 was found to cause significant off-target activity in the expressed RNA sequences of edited human cells with a frequency ranging between 0.07% and 100%.^64^ Secondly, a study quantitatively evaluating RNA single nucleotide variants caused by base editing demonstrated that both CBE and ABEs induced thousands of off-target conversions.^65^ Moreover, transfection of the single APOBEC1 or TadA enzymes produced higher rates of off-targets, indicating that these enzymes are the most likely the cause of the effect, consistent with a previous report.^62^

## Optimization of the base editors

Base editing is proving to be an extensively adaptable tool for laboratory animal research and may avoid some of undesirable consequences associated with the DSBs introduced when adopting a classical CRISPR editing approach. Although there are conflicting reports concerning the accuracy of base editing, it is clear that unwanted mutations or indels are still associated with this technique. The precision required for single base conversion has thus led to a widespread focus on the improvement of both CBE and ABEs and many optimizations addressing accuracy and specificity of the editors have been reported^66^. We summarize the key developments, focussing on those tested in animal models.

Firstly, with respect to target range, a major limitation in the use of base editing systems is simply the lack of an available CRISPR target site. The relative positioning of the target nucleotide, the activity window and a suitable PAM sequence frequently prohibits the use of Cas9-based editing systems. To overcome these limitations, several teams have produced base editors with either different Cas enzymes and Cas9 orthologues such as *Staphylococcus aureus* Cas9, Cas12a or mutated versions of the same SpCas9 recognizing different PAM sequences.^67,68^ The targeting range of BE3 has been expanded by introducing PAM-altering mutations into the SpCas9 sequence, generating VQR-BE3 (NGA PAM), EQR-BE3 (NGAG PAM), VRER-BE3 (NGCG PAM) and SAKKH-BE3 (NNRRT PAM).^68^ These new BE3 systems demonstrated good editing efficiency at the new target sites, and SAKHH-BE3 has been applied to induce base conversion in human zygotes,^69^ whereas VQR-BE3 showed significantly higher editing efficiency than BE3 in mouse embryos.^57^ Similarly, directed evolution of SpCas9 has given rise to xCas9, characterised by a NGN or GAA/GAT PAM and thus equipped with an expanded targeting range.^70^ Directed mutation of SpCas9 has also resulted in Cas9-NG, a version with a very flexible NG PAM, which was functional when incorporated within a Target-AID base editor.^71^

Optimization of base editor tools has also been focussed on increasing the editing precision by decreasing the size of the activity window through modification of the APOBEC component. David Liu and co-workers generated a number of BE3 iterations aimed at maximising the base editor’s precision by narrowing down the activity window to only ∼1–2nt.^68^ These YE1-BE3, YE2-BE3, EE-BE3 and YEE-BE3 iterations lowered the risk of unwanted conversion of non-target nucleotides. Similarly, the classic rat APOBEC1 has been exchanged for the human engineered APOBEC3A(A3A), generating the A3A-BE3, which, when purified as an RNP and electroporated into human erythroid precursors, led to successful editing of mutations implicated in β-thalassemia.^72^

A fourth generation CBE (BE4) has been developed, aimed at improving efficiency and avoiding unwanted G–C or A–T conversions. It was determined that the UNG repair mechanism was likely the main cause for unwanted conversions when not completely blocked by the UGI inhibitor. Therefore, BE4 was generated by adding additional UGI copies and increasing the size of the linkers connecting each component, allowing increased flexibility.^73^ Moreover, it was discovered that fusion of Gam, a bacteriophage protein that binds to DSBs, to BE4, generated significantly fewer indels. Although BE4 did not show substantial improvements compared with BE3 when editing sequences in zygotes, BE4-Gam was shown to be highly effective for the generation of base-edited rabbits.^29^ This lead to further iterations of both CBE and ABE (BE4max and ABEmax) characterised by codon optimisations and improved nuclear localisation signals and the same study reported an optimized CBE, AncBE4max, with an ancestral reconstruction of the deaminase component.^74^ BE4max and AncBE4max have been applied successfully in zebrafish embryos, with considerable improvements in efficiency and precision when compared with BE3 and Target-AID.^75^ AncBE4max has also been used recently to generate loss-of-function pig models by zygote microinjection, and showed considerable efficiency improvements when compared with BE4-Gam, although also inducing a higher frequency of bystander edits.^33^ BE4max has also been used to generate a monkey model of Hutchinson–Gilford progeria syndrome, editing the pathogenic human mutation (Gly608Gly) at the *LMNA* gene by microinjection of cynomolgus monkey zygotes.^76^ Five out of the six live offspring resulting from the experiment harboured the desired mutation, three of which were homozygous, expressing phenotypes that clearly mimicked the disorder in humans and thus demonstrating a very high efficiency for this optimized CBE. Similarly, high efficiency for the optimized ABE editor, ABEmax, has also been reported in large animals models, with successful introduction of the Booroola fecundity mutation at the *BMPR1B* gene in sheep.^77^

Optimization of the BE4-Gam construct design by removing sites causing premature polyadenylation and further codon and nuclear localisation optimisation, has been shown to improve editing efficiency significantly in both organoids and mouse models.^78^ Modifications from BE4max and the YE1-BE3 system have been combined and further refined, increasing the precision of the resulting YFE-BE4max,^79^ which was used to generate albino and prematurely aged rabbits by knocking out the *Tyr* and the *Lmna* genes, respectively. Notably, all the founder rabbits were found to be homozygous for the *Tyr* knock-out and thus showed a clear albino phenotype. And, although additional mutations were noted (<15%), they were significantly lower than those found by BE3 editing.^29^ Modifications from ABEmax and NG-Cas9 have been combined to generate a new ABE iteration that was proven capable of editing different sites in mouse embryos with a significantly expanded editing range compared with ABEmax.^80^

In order to overcome off-target mutations in the transcriptome, David Liu’s team investigated possible solutions to minimize this unwanted phenomenon.^64^ A mutated APOBEC1 (R33A/K34A) was found to have the same efficiency of the normal BE3 but drastically reduced the off-target rate in the transcriptome. A similar outcome of reduced RNA editing and improved specificity resulted from the incorporation of a E59A mutation in the wild-type TadA and an V106W substitution in the mutant TadA*, creating ABEmaxAW.^81^

Finally, one of the main disadvantages of both ABE and CBE has been their large size, which forces their split delivery between two AAV vectors. Although overcoming the size limitation, this does impact the resulting editing efficiency.^43,44,50^ A recent study reported the development of split-intein base editors, facilitating the reconstitution of the system when delivered through two different AAV vectors.^82^ The efficiency of the new split-intein ABE and CBE was tested in mouse tissues, such as brain, liver, retina, heart and skeletal muscle, and achieved editing efficiencies ranging from 9% to 59%. These results demonstrate the significant improvement of ABE and CBE delivery systems for somatic cells, facilitating applications of base editors as gene therapy treatments.

A number of base editor systems have recently been described, which combine the functionality of both CBE and ABEs in a single enzyme. Three independent groups fused TadA heterodimers (from ABE7.10 or subsequent iterations) to a Target-AID like CBE structure, generating enzymes, Target-ACEmax,^83^ A&C-BEmax and SPACE,^84,85^ with the attributes of both constituent CBE and ABE components. These enzymes showed both single (CBE or ABE) activity, but could also be used for simultaneous C-to-T and A-to-G edits at a target site. Specificity and accuracy were found to be very similar to the parental enzymes.^83–85^ Although not yet tested in animal models, these enzymes have the potential to provide further flexibility for the introduction of targeted genomic changes within both embryos and somatic tissue in vivo.

The development and optimization of base editors is fast paced, and a whole suite of improvements are quickly being developed to tackle any undesired effects, leading to substantial improvements and the production of base editors with ever increasing precision and target range.^66^ When applied in animals, these continual developments will also positively impact welfare by decreasing the risk of unwanted mutations, thus refining the manipulations in line with the principles of the 3Rs.

## Prime editing

**Figure 4.**
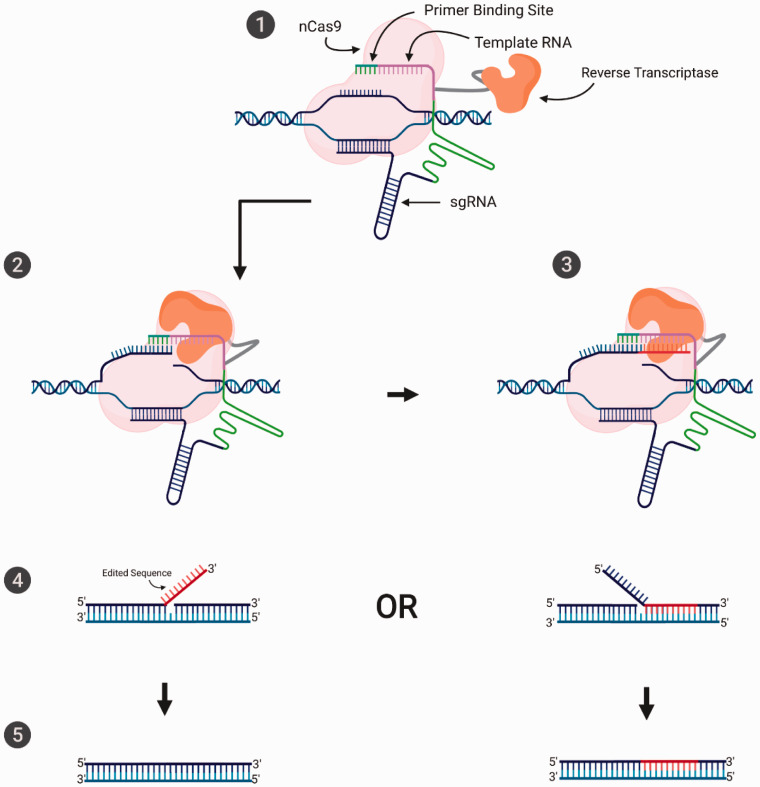
The molecular mechanism of prime editing. The prime editing machinery, composed of a H840A nCas9 fused to a reverse transcriptase is directed to the target sequence by a pegRNA, a sgRNA fused to a template RNA and primer binding site (1). At the target site, the nCas9 separates the two strands while nicking the non-target strand, which is now free to bind to the primer binding site (2). The reverse transcriptase elongates the non-target strand using the template RNA (containing the required mutation) (3). The non-target strand now contains an ssDNA that does not match the target strand. When the two strands hybridise again, the excess nucleotides form a ‘flap’. Two possible flaps can be generated through this event. Either the strands hybridise matching as before, thus leaving the newly edited sequence outside or the opposite occurs leaving the non-edited sequence as the ‘flap’ (4). The flap is excised by the FEN1 endonuclease. An additional nick can be introduced on the unedited strand (not shown on the figure for clarity), which can stimulate its repair using the edited sequence, effectively completing the editing on both strands (5).

Although base editing represents a considerable innovation in the field of genome engineering, it is still neither able to tackle all base conversions, nor insert or eliminate larger sections of DNA – a function still very much needed to not only generate and study genetic aberrations in model animals but also to develop therapeutic tools addressing diverse genetic disorders. For this reason, the same laboratory that invented both CBE and ABE developed a new tool, coined prime editing.^86^ Like its predecessor, prime editing is a system generated by equipping the Cas9 enzyme with new catalytic domains. The tool is composed of a Cas9 nickase (H840A mutation, Figure 2c) fused to a reverse transcriptase domain, together with a modified sgRNA, named prime editing guide RNA (pegRNA). The elegance behind the engineering of this editor lies within the pegRNA, which is composed of the classic sgRNA, guiding the Cas9 to the correct target site while simultaneously harbouring the sequence that will modify the DNA. Guided by the pegRNA, the H840A nCas9 nicks the non-target strand, exposing a 3′ hydroxyl group, where the reverse transcriptase inserts the new DNA sequence using the information encoded within the pegRNA. As the added sequence causes a redundancy of nucleotides, the DNA will repair itself resulting in the generation of two ‘flaps’. Either a 5′ flap containing the unedited strand of DNA while the new sequence has been inserted in the genome, or a 3′ flap containing the new reverse transcribed sequence while the original DNA sequence has been inserted. However, as the repair intermediate tends to eliminate redundant DNA and structure specific endonucleases such as FEN1 have a bias preference for the excision of 5′ flaps, editing will be biased for elimination of the 5′ unedited strand, resulting in the incorporation of the edited sequence (Figure 4). The desired edited outcome can be encouraged by the nicking of the non-edited strand (between 14 and 116 nt from the editing site) or after the editing has occurred by using a sgRNA targeting the new sequence in order to avoid further nicking if the editing has not occurred correctly. The system was demonstrated to be able to carry out both small (between 1 and 3nt) and large (between 5 and 80nt) insertion and deletions with relatively low indel rates. Finally, in this initial study, the targeted use of prime editing for the treatment of sickle cell anaemia in HEK293T resulted in 44% efficiency with a relatively low 4.8% indel rate.

Recent publications have started to explore the functionality of prime editing in model organisms. Functionality has been confirmed in rice,^87^ and, in animals, the first application in mice has yielded positive results.^88^ PE3 mRNA and a pegRNA targeting the *Hoxd13* gene was microinjected into mouse embryos. Two different *Hoxd13* sites were chosen, the first targeting a G-to-C conversion and the latter G-to-T. For these two target sites, nucleotide conversions was observed in 27% and 10.5% of the founder mice, respectively. Interestingly, the indel rate ranged from 0% to 0.3%; however, a series of unwanted conversions was also noted within the activity window. Of note, a recent study has also successfully achieved prime editing of the GFP gene in induced pluripotent stem cells.^89^ The study reported that successful editing was influenced by the size of the pegRNA; prime editing with an 11 nt pegRNA resulted in 6.5% editing efficiency, whereas 15 nt pegRNA resulted in an editing rate of only 0.3%.

Similarly to what had been already observed for base editors, these initial experiments on prime editing highlight its feasibility for the generation of model organisms while also suggesting a lower level of precision. These early studies will no doubt encourage future iterations and optimization, to address shortcomings. Furthermore, an application in the field of gene therapy is anticipated.

## Conclusion

This review has assessed the development of base and prime editing and its application in the field of animal research. Many studies have confirmed that these tools are useful for the generation of animal models harbouring precise mutations. The key advantage of the technologies is that they do not rely on the introduction of a DSB at the target site – a lesion that has been associated with non-specific DNA damage in conventional CRISPR editing approaches. Moreover, through the high editing efficiency of these systems, several animal models have been generated with homozygous mutations in the founder generation, resulting in significant savings in both animal numbers and improvements in time and experimental cost. Furthermore, the base editors’ ability to target single nucleotides is allowing the development of new gene therapy tools, frequently involving viral AAV delivery, opening the prospect of treatment of a multitude of pathogenic mutations in humans. However, it is important to note that these tools show imperfections affecting their editing precision – a key necessity before ABE and CBE tools can be used in the clinic. In response to these limitations, extensive innovation and development has produced new iterations aimed at resolving these deficiencies. Finally, the invention of prime editing has opened up new possibilities, enabling larger sequences to be inserted or deleted without introducing a DSB, thus addressing the niche that base editors have not been able to address. An interesting question emerges of whether prime editing will supersede base editing. Although this question is currently impossible to answer, it is clear that the efficiencies and accuracies of diverse applications of the two technologies will need to be assessed to allow the answer to be determined. It will also be interesting to evaluate the amount of optimisation that each editing system requires and further precision of design rules, especially for prime editing, will be an important area of research. For the foreseeable future, however, continued application of both base editors and a new application of prime editors in animal research will allow continued improvements in the generation of animal models and testing of gene therapy approaches.
